# β-hydroxybutyrate reduces blastocyst viability via trophectoderm-mediated metabolic aberrations in mice

**DOI:** 10.1093/humrep/deac153

**Published:** 2022-07-20

**Authors:** Emma G Whatley, Thi T Truong, Dagmar Wilhelm, Alexandra J Harvey, David K Gardner

**Affiliations:** School of BioSciences, University of Melbourne, Parkville, Victoria, Australia; School of BioSciences, University of Melbourne, Parkville, Victoria, Australia; Department of Anatomy & Physiology, University of Melbourne, Parkville, Victoria, Australia; School of BioSciences, University of Melbourne, Parkville, Victoria, Australia; School of BioSciences, University of Melbourne, Parkville, Victoria, Australia; Melbourne IVF, East Melbourne, Victoria, Australia

**Keywords:** beta-hydroxybutyrylation, DOHaD, embryo transfer, epigenetics, ketogenic diet, ketone, metabolism, morphokinetics, nutrients

## Abstract

**STUDY QUESTION:**

What is the effect of the ketone β-hydroxybutyrate (βOHB) on preimplantation mouse embryo development, metabolism, epigenetics and post-transfer viability?

**SUMMARY ANSWER:**

*In vitro* βOHB exposure at ketogenic diet (KD)-relevant serum concentrations significantly impaired preimplantation mouse embryo development, induced aberrant glycolytic metabolism and reduced post-transfer fetal viability in a sex-specific manner.

**WHAT IS KNOWN ALREADY:**

A maternal KD in humans elevates gamete and offspring βOHB exposure during conception and gestation, and in rodents is associated with an increased time to pregnancy, and altered offspring organogenesis, post-natal growth and behaviour, suggesting a developmental programming effect. *In vitro* exposure to βOHB at supraphysiological concentrations (8–80 mM) perturbs preimplantation mouse embryo development.

**STUDY DESIGN, SIZE, DURATION:**

A mouse model of embryo development and viability was utilized for this laboratory-based study. Embryo culture media were supplemented with βOHB at KD-relevant concentrations, and the developmental competence, physiology, epigenetic state and post-transfer viability of *in vitro* cultured βOHB-exposed embryos was assessed.

**PARTICIPANTS/MATERIALS, SETTING, METHODS:**

Mouse embryos were cultured *in vitro* with or without βOHB at concentrations representing serum levels during pregnancy (0.1 mM), standard diet consumption (0.25 mM), KD consumption (2 mM) and diabetic ketoacidosis (4 mM). The impact of βOHB exposure on embryo development (blastocyst formation rate, morphokinetics and blastocyst total, inner cell mass and trophectoderm (TE) cell number), physiology (redox state, βOHB metabolism, glycolytic metabolism), epigenetic state (histone 3 lysine 27 β-hydroxybutyrylation, H3K27bhb) and post-transfer viability (implantation rate, fetal and placental development) was assessed.

**MAIN RESULTS AND THE ROLE OF CHANCE:**

All βOHB concentrations tested slowed embryo development (*P *<* *0.05), and βOHB at KD-relevant serum levels (2 mM) delayed morphokinetic development, beginning at syngamy (*P *<* *0.05). Compared with unexposed controls, βOHB exposure reduced blastocyst total and TE cell number (≥0.25 mM; *P *<* *0.05), reduced blastocyst glucose consumption (2 mM; *P *<* *0.01) and increased lactate production (0.25 mM; *P *<* *0.05) and glycolytic flux (0.25 and 2 mM; *P *<* *0.01). Consumption of βOHB by embryos, mediated via monocarboxylate transporters, was detected throughout preimplantation development. Supraphysiological (20 mM; *P *<* *0.001), but not physiological (0.25–4 mM) βOHB elevated H3K27bhb levels. Preimplantation βOHB exposure at serum KD levels (2 mM) reduced post-transfer viability. Implantation and fetal development rates of βOHB-treated embryos were 50% lower than controls (*P* < 0.05), and resultant fetuses had a shorter crown-rump length (*P *<* *0.01) and placental diameter (*P *<* *0.05). A strong sex-specific effect of βOHB was detected, whereby female fetuses from βOHB-treated embryos weighed less (*P *<* *0.05), had a shorter crown-rump length (*P *<* *0.05), and tended to have accelerated ear development (*P *<* *0.08) compared with female control fetuses.

**LIMITATIONS, REASONS FOR CAUTION:**

This study only assessed embryo development, physiology and viability in a mouse model utilizing *in vitro* βOHB exposure; the impact of *in vivo* exposure was not assessed. The concentrations of βOHB utilized were modelled on blood/serum levels as the true oviduct and uterine concentrations are currently unknown.

**WIDER IMPLICATIONS OF THE FINDINGS:**

These findings indicate that the development, physiology and viability of mouse embryos is detrimentally impacted by preimplantation exposure to βOHB within a physiological range. Maternal diets which increase βOHB levels, such as a KD, may affect preimplantation embryo development and may therefore impair subsequent viability and long-term health. Consequently, our initial observations warrant follow-up studies in larger human populations. Furthermore, analysis of βOHB concentrations within human and rodent oviduct and uterine fluid under different nutritional states is also required.

**STUDY FUNDING/COMPETING INTEREST(S):**

This work was funded by the University of Melbourne and the Norma Hilda Schuster (nee Swift) Scholarship. The authors have no conflicts of interest.

**TRIAL REGISTRATION NUMBER:**

N/A.

## Introduction

The ketogenic diet (KD), characterized by very high fat and low carbohydrate consumption, was first reported in 1921 for the treatment of drug-resistant epilepsy (Wilder, 1921; Hohn *et al.*, 2019). Subsequent studies have revealed that a KD can be beneficial in treating Alzheimer’s and Parkinson’s disease (Veech *et al.*, 2001), type 2 diabetes (Hussain *et al.*, 2012), obesity (Murphy and Jenkins, 2019) and cancer (Seyfried *et al.*, 2003; Morscher *et al.*, 2015; Klement, 2017). More recently, interest has risen in using the KD to aid infertility, specifically, polycystic ovary syndrome (PCOS); the leading cause of anovulatory infertility that is tightly associated with hormonal and metabolic dysregulation (March *et al.*, 2010; Muscogiuri *et al.*, 2016). Evidence suggesting a KD contributes to hormonal re-balancing (by reducing the LH/FSH ratio and free testosterone, and increasing progesterone) and insulin re-sensitization (Boden *et al.*, 2005; Mavropoulos *et al.*, 2005; Gupta *et al.*, 2017; McGrice and Porter, 2017; Paoli *et al.*, 2020), and consequently reverses amenorrhoea and anovulation, indicates that a KD may benefit the fertility of women with PCOS (Mavropoulos *et al.*, 2005; Alwahab *et al.*, 2018; Paoli *et al.*, 2020). In addition, this diet is also gaining popularity amongst otherwise healthy women trying to conceive. The effect of a KD on human reproduction and offspring health is unknown, though limited mouse studies have indicated that KD consumption for 30 days prior to- and during gestation reduces female fertility by increasing the time to pregnancy while altering fetal neuroanatomy and postnatal/adult behaviour (Sussman *et al.*, 2013a,b, 2015). These data infer that a KD could significantly alter the programming of gamete, embryo and/or fetal development.

A KD induces a physiological switch to fatty-acid rather than carbohydrate-based metabolism, which significantly elevates serum concentrations of the ketones β-hydroxybutyrate (βOHB), acetoacetate (AcAc) and acetone. Whereas acetone is primarily expelled via the lungs (Anderson, 2015), serum AcAc and βOHB are highly effective oxidative fuels for cells, with βOHB being the predominant ketone that is maintained at two to three times the concentration of AcAc. Compared with a standard diet, a KD elevates serum βOHB concentrations by 10- to 18-fold from ∼0.12 mM to 2.09 mM in humans, and from ∼0.23 mM to 2.43 mM in rodents (Table I). Whether this leads to a corresponding elevation of βOHB within the oviduct and uterine fluids is yet to be determined. However, maternal diet is known to influence the nutrient composition of female reproductive tract fluid (Kermack *et al.*, 2015), and βOHB is a short-chain carboxylic acid with high vascular permeability that is readily transported across plasma membranes via monocarboxylate transporters (MCTs) (Halestrap, 1978; Laffel, 1999; Halestrap and Wilson, 2012; Halestrap, 2013). These transporters are present on embryonic cells and other reproductive tissues including the ovary, ciliated cells within the ampulla and isthmus regions of the oviduct, and the uterine surface epithelium and glands (Gardner and Leese, 1988; Harding *et al.*, 1999; Jansen *et al.*, 2008; Kuchiiwa *et al.*, 2011). Indeed, ketones have been detected in human follicular fluid (Piñero‐Sagredo et al., 2010) and human amniotic fluid (Kim and Felig, 1972) at concentrations positively correlated with maternal serum ketone levels. Therefore, βOHB may accumulate within reproductive fluid, increasing its availability and consumption by the embryo, and have the potential to affect embryo development. However, the concentration of βOHB in oviduct and uterine fluids and its impact on embryo development requires confirmation.

**Table I:** Concentrations of the ketone β-hydroxybutyrate (βOHB) in human and rodent plasma and used in the *in vitro* embryo culture medium in this study (*in vitro* (G1/G2)), representative of levels during pregnancy, consumption of a standard diet, a ketogenic diet, or diabetic ketoacidosis.

Nutritional state	βOHB (mM)	References
**Pregnancy**		

Human	0.12	Kim and Felig (1972), Jovanovic *et al.* (1998), Bon *et al.* (2007), Haruna *et al.* (2010)
Rodent	0.13	Kervran *et al.* (1978), Villarroya and Mampel (1985)
*In vitro* (G1/G2)	0.1	

**Standard diet**		

Human	0.12	Tanda *et al.* (2014), White *et al.* (2017)
Rodent	0.23	Senior and Sherratt (1969), Ruderman *et al.* (1974), Lemieux *et al.* (1984), Villarroya and Mampel (1985), Thio *et al.* (2006)
*In vitro* (G1/G2)	0.25	

**Ketogenic diet**		

Human	2.09	Garber *et al.* (1974), Reichard *et al.* (1979)
Rodent	2.43	Senior and Sherratt (1969), Ruderman *et al.* (1974), Lemieux *et al.* (1984), Villarroya and Mampel (1985), Thio *et al.* (2006)
*In vitro* (G1/G2)	2	

**Diabetic ketoacidosis**		

Human	3.98	Siegel *et al.* (1977), Okuda *et al.* (1996)
Rodent	5.15	Ruderman and Goodman (1974), Lemieux *et al.* (1984), Silver *et al.* (1997)
*In vitro* (G1/G2)	4	

Embryos respond to changes in environmental nutrient availability by undergoing metabolic (Menke and McLaren, 1970; Lane and Gardner, 2000, 2003; Gardner and Wale, 2013) and epigenetic (Doherty *et al.*, 2000; Market-Velker *et al.*, 2010, 2012) adaptations. While such plasticity enables short-term survival adaptation, the persistent nature of such changes can be harmful by predisposing offspring to long-term developmental programming and health issues. This phenomenon, emphasized by the developmental origins of health and disease (DOHaD) (Barker and Osmond, 1986; Hales and Barker, 1992; Fleming *et al.*, 2015), may be underpinned mechanistically by the interrelationship between metabolism and epigenetic state, termed ‘metaboloepigenetics’ (Donohoe and Bultman, 2012; Harvey *et al.*, 2016; Harvey, 2019).

Post-compaction, the embryo relies heavily on glycolytic metabolism to support the production of biosynthetic precursors, cytosolic redox state, and to ensure lactate efflux for maternal-embryo signalling during implantation (Hewitson and Leese, 1993; Gardner, 1998; Harvey *et al.*, 2002; Gardner, 2015; Gardner and Harvey, 2015; Ma *et al.*, 2020; Gurner *et al.*, 2022). However, βOHB promotes oxidative phosphorylation (Laffel, 1999) while suppressing glycolysis (Randle *et al.*, 1964; Patel and Owen, 1977; Hunter and Sadler, 1987; Cox *et al.*, 2016). This is of significance given that aberrant glucose uptake and glycolytic metabolism is a well-characterized biomarker of poor embryonic viability (Lane and Gardner, 1996; Gardner and Wale, 2013). βOHB also acts directly as an epigenetic modifier, via class I histone deacetylase (HDAC) inhibition (Newman and Verdin, 2014) and up-regulation of histone lysine β-hydroxybutyrylation (Kbhb) (Xie *et al.*, 2016; Sangalli *et al*., 2022), and could feasibly impair the intricate chromatin programming that is characteristic of, and integral to, successful pre- and post-implantation development at a cost to long-term developmental and health outcomes.

Rodent studies have revealed a KD deleteriously affects female fertility by increasing time to pregnancy and reducing litter sizes (Sussman *et al.*, 2013a). A pre-conception and gestational KD induced mouse fetal organ growth alterations, including to the heart, pharynx, spinal cord and brain, which may lead to organ dysfunction (Sussman *et al.*, 2013a,b, 2015). Notably, pups from KD consuming mothers that were standard fed postnatally exhibited growth retardation and neurofunctional and behavioural alterations including reduced susceptibility to anxiety and depression and increased hyperactivity (Sussman *et al.*, 2013a, 2015; Wojciech *et al.*, 2022). While these latter findings may be interpreted as a beneficial effect, they also infer a significant developmental programming effect of a gestational KD on offspring neurodevelopment and behaviour. Similarly, *in vitro* studies have demonstrated exposure to high levels of βOHB (8 to 80 mM) in culture medium retards preimplantation embryo development compared to untreated controls (Zusman *et al.*, 1987; Moley *et al.*, 1994). However, the findings of these *in vitro* studies are limited due to their use of high βOHB concentrations and assessment of embryo morphology alone. More in-depth analysis of perturbations during preimplantation development, including mechanistic investigations into the physiology underpinning the developmental aberrations that may contribute to developmental programming, are therefore required.

This study aimed to determine whether exposure of preimplantation embryos to βOHB at physiologically relevant concentrations could program offspring development and health, by examining its impact on mouse embryo development, physiology, epigenetic state and post-transfer viability.

## Materials and methods

### Animals and superovulation

Mice were housed under a 12 h light, 12 h dark photoperiod, with standard mouse chow (Barastoc GR2 Rat & Mouse Maintenance Cube, 13.5 MJ/kg energy from 3% fat, 20% protein, 75% carbohydrate) and water available *ad libitum*. Three- to 4-week-old virgin F1 female mice (C57BL/6 × CBA) were superovulated by intraperitoneal injection of 5 IU pregnant mare’s serum gonadotropin (PMSG; Folligon, Intervet, UK) in the middle of the light photoperiod, followed 48 h later by 5 IU human chorionic gonadotropin (hCG; Chorulon, Intervet, UK). Females were housed overnight with a single male (>12 weeks old) of the same strain, and successful mating was confirmed by the presence of a vaginal plug the following morning.

### Embryo collection

Pronucleate oocytes (2PN) were collected 21 h post-hCG in warmed (37°C) GMOPS PLUS (Vitrolife AB, Sweden) handling medium containing human serum albumin (HSA, 5 mg/ml; Vitrolife) (Gardner and Lane, 2014; Gardner and Truong, 2019), and exposed to GMOPS PLUS containing 300 IU/ml hyaluronidase (bovine testes type IV, Sigma Aldrich, NSW, Australia) to denude cumulus cells. Pronucleate oocytes from multiple mice were subsequently pooled and washed twice in GMOPS PLUS, and once in G1 medium (Gardner and Truong, 2019) containing HSA (5 mg/ml) before random allocation to treatments.

### Embryo culture and treatments

Embryos were cultured in G1 medium containing HSA (5 mg/ml) for 47.5 h, then transferred to G2 medium with HSA (5 mg/ml) for the remainder of culture unless otherwise specified. Fresh culture media were prepared in-house monthly. Culture media were supplemented without (control) or with D, L-sodium βOHB (Sigma-Aldrich), at concentrations representative of serum levels with pregnancy (0.1 mM), consumption of a standard diet (0.25 mM), nutritional ketosis/KD consumption (2 mM) or diabetic ketoacidosis (4 mM) (Table I), or at concentrations otherwise specified. The addition of βOHB to media did not alter osmolarity or pH. Embryos were cultured in groups of 10 in 20 μl drops of medium under paraffin oil (Ovoil, Vitrolife) in a humidified multi-gas incubator (MCO-5M[RC], Sanyo Electric, Osaka, Japan) at 37°C, 6% CO_2_, 5% O_2_ and 89% N_2_. For all cultures, 35 mm petri dishes (Falcon, BD Biosciences, NJ, USA) were used, unless specified otherwise. Embryos cultured in groups were assessed for developmental rates until the blastocyst stage, blastocyst cell number, glycolytic metabolism, βOHB metabolism, NAD(P)H autofluorescence, histone β-hydroxybutyrylation, and post-transfer viability and development (embryo transfers).

### Differential cell allocation in the blastocyst

Day 5 expanded, hatching and fully hatched blastocysts were selected for differential nuclear staining at 121 h post-hCG to assess the number of inner cell mass (ICM) and trophectoderm (TE) cells (Hardy *et al.*, 1989). Pronase was used to remove the zona, after which 10 mM trinitrobenzenesulphonic acid, 0.1 mg/ml anti-dinitrophenol and guinea pig complement serum containing 0.1 mg/ml propidium iodide were utilized to label TE cells, while the ICM cells remained unlabelled. All cells were counterstained in 0.1 mg/ml bisbenzimide (Hoechst 33258). Blastocysts were mounted on glass microscope slides in 100% (v/v) glycerol and imaged on a Nikon Eclipse Ts100 inverted fluorescent microscope equipped with a Nikon digital sight DS-Fi camera. FIJI image analysis software (ImageJ 1.52a, https://imagej.net/software/fiji/) was used to count cell numbers.

### Morphokinetic analysis

Embryos were cultured individually from the 2PN to blastocyst stage in 25 μl drops of G1 medium with HSA (48 h) and G2 medium with HSA (subsequent 48 h) in an EmbryoScope™ time-lapse imaging incubator (Vitrolife). Media were supplemented with or without 2 mM βOHB, and embryos were cultured in EmbryoSlide (Vitrolife) culture dishes. Time-lapse images were captured every 15 min, and key developmental events recorded. Given that fertilization occurred *in vivo* and therefore its timing could not be accurately determined, morphokinetic events were measured as both hours post-hCG injection and hours post 2-pronuclei fading (tPNF). Developmental time-points analysed included t2, t3, t4, t5, t6, t7, t8, tM, tSB, tB, tEB and tHB, which respectively represented time to cleavage to 2-cell, 3-cell, 4-cell, 5-cell, 6-cell, 7-cell, 8-cell, morulae, start of blastocoel formation, blastocyst, expanded blastocyst and hatching blastocyst (Gardner and Truong, 2019). Individually cultured embryos were assessed for morphokinetic development only, but no other experimental endpoints.

### Analysis of βOHB metabolism

Embryos were cultured for 6 or 8 h, as specified, in groups of 5 in 500 nl drops of metabolic G1 (2PN and 2-cell) or metabolic G2 (compacting/morula, Day 4 blastocyst, Day 5 blastocyst). Metabolic media were devoid of glucose, lactate or pyruvate and were supplemented with 0.5–8 mM βOHB, with or without the MCT1 and MCT2 inhibitor, α-cyano-4-hydroxycinnamate (CHC, 0.125 mM). The optimal concentration of CHC was determined by evaluating the effectiveness of 0.125–1 mM CHC on the inhibition of βOHB uptake by 8 mM βOHB-exposed Day 5 blastocysts (Supplementary Fig. S1).

Embryonic βOHB metabolism was assessed via ultramicrofluorescence (UMF), which utilizes enzymatic reactions coupled to the fluorescent pyridine nucleotide NADH (Leese and Barton, 1984; Gardner and Leese, 1990). The βOHB assay for UMF was specifically developed for this study and was a miniaturized and modified version of previously used protocols (Hansen and Freier, 1978; Li *et al.*, 1980; Nuwayhid *et al.*, 1988; Christopher *et al.*, 1992), utilizing the below reaction:

βOHB + NAD+→β-hydroxybutyrate dehydrogenase 1(3-BDH1)AcAc + NADH + H+

Hand-made, calibrated glass volumetric constriction micropipettes (Mroz and Lechene, 1980; Gardner, 2007) were used to add nanolitre volumes of spent media samples to the βOHB assay (60 mM Tris-HCl buffer, 14 mM NAD^+^, 1.5 U/ml βOHB dehydrogenase (EC 1.1.1.30), pH 9.5). The reaction was incubated in the dark for 10 min under paraffin oil on a siliconized microscope slide at 37°C, then cooled to room temperature for 5 min prior to imaging NADH fluorescence, indicative of βOHB concentration. βOHB concentration was quantified by comparison to a 6-point standard curve (*R*^2^ > 0.99), and embryonic βOHB utilization determined by comparison to an unspent (no embryo) control medium sample.

### Analysis of glycolytic metabolism

Day 5 blastocysts were cultured individually in 500 nl metabolic G2 (0.5 mM glucose, no lactate or pyruvate) with or without 0.25–8 mM βOHB for 6 or 8 h as specified. Blastocyst glucose and lactate metabolism of individual embryos was quantitated via UMF (Leese and Barton, 1984; Gardner and Leese, 1990).

Media samples were added to the glucose assay (0.42 mM dithiothreitol (DTT), 3.09 mM magnesium sulfate (MgSO_4_), 0.42 mM adenosine triphosphate (ATP), 1.25 mM NADP^+^, 14.11 U/ml hexokinase (EC 2.7.1.1), 7.06 U/ml glucose-6-phosphate dehydrogenase (EC 1.1.1.49) in EPPS buffer, pH 8.0) or to the lactate assay (4.76 mM NAD^+^, 195.3 U/ml lactate dehydrogenase (EC 1.1.1.27) in glycine hydrazine buffer, pH 9.4). The reaction was incubated for 10 min under paraffin oil on a siliconized microscope slide at 37°C, then allowed to cool to room temperature in the dark for 2 min prior to imaging NAD(P)H fluorescence, indicative of carbohydrate concentration which was determined by comparison to a 6-point standard curve (*R*^2^ > 0.99). Blastocyst glucose consumption and lactate production was determined by comparison to a co-incubated unspent (no embryo) control medium sample. Glycolytic flux (%) was determined on the basis that 1 mol glucose produces 2 mol lactate.

### NAD(P)H autofluorescence

Embryos were transferred into a glass-bottomed imaging dish (FluoroDish, World Precision Instruments, Inc., Hilton, SA, Australia), washed three times in treatment medium, and cultured individually in 500 nl drops of G2 or metabolic G2 medium (no glucose, lactate or pyruvate) with or without 2 mM βOHB for 20 min prior to imaging. On a heated stage in the dark, embryos were imaged once under a 4′,6-diamidino-2-phenylindole (DAPI) filter (500 ms exposure, ×200 magnification, excitation wavelength: 360 nm, emission wavelength: 435–485 nm), with a Nikon Eclipse Ti-U inverted microscope equipped with a Photometrics Coolsnap HQ_2_ camera with NIS Elements BR 3.0 software (Nikon Instruments Inc.). The NAD(P)H autofluorescence intensity was quantified using FIJI image analysis software. The total area of each embryo was selected by tracing its perimeter, in addition to three spaces of background area, and the area, integrated density and mean fluorescence of each selection was calculated. The corrected total cellular fluorescence (CTCF) was calculated for each embryo, with equalization to background fluorescence (CTCF = [embryo integrated density] − [area of embryo × averaged mean fluorescence of background readings]) (McCloy *et al.*, 2014).

### Immunofluorescence for analysis of Kbhb

Immunofluorescent staining of blastocysts was conducted as described previously (Harvey *et al.*, 2004). The zona pellucida of Day 4 blastocysts was removed by brief (<10 s) exposure to warmed (37°C) Tyrode’s solution, acidic (Sigma). Zona free embryos were then washed three times in pre-warmed (37°C) GMOPS PLUS, and once in G2 medium before being returned to culture. Day 5 blastocysts were fixed 24 h after zona removal in 4% (v/v) paraformaldehyde (PFA) in phosphate buffered saline (PBS) for 10 min and stored in 0.4% (v/v) PFA in PBS under mineral oil at 4°C for up to 1 month. Blastocysts were washed once in blocking solution (0.5% (w/v) bovine serum albumin, 1% (v/v) Tween 20 in PBS) for 5 min, permeabilized for 15 min in 0.25% (v/v) Triton X-100 in PBS, neutralized in 50 mM NH_4_Cl (Sigma) for 10 min, and blocked for non-specific binding for at least 1 h at room temperature in blocking solution supplemented with 5% (v/v) donkey serum (Sigma-Aldrich). Blastocysts were exposed to mouse α CDX2 (Abcam, ab157524, 1:50 dilution) overnight in a humidified chamber at 4°C, then incubated in donkey α mouse Alexa Fluor 488 (Invitrogen, A21202, 1:2000 dilution) for 50 min at room temperature in a humidified chamber, in the dark. Blastocysts were blocked for a further 2 h at room temperature using blocking solution with 5% (v/v) donkey serum, and subsequently incubated overnight in rabbit α histone 3 lysine 27 β-hydroxybutyrylation (H3K27bhb) (Abcam, ab241463, 1:100 dilution) at 4°C in a dark humidified chamber. Donkey α rabbit Alexa Fluor 568 (Life Technologies, A10042, 1:2000 dilution) secondary antibody was applied for 50 min at room temperature in a humidified chamber in the dark. Embryos were washed three times for 10 min and 20 min each after incubation in primary and secondary antibodies, respectively. Blastocysts were counterstained in 0.1 mg/ml bisbenzimide for 7 min and mounted on glass microscope slides in 100% (v/v) glycerol under coverslips. Negative controls consisted of embryos stained without primary antibodies, or with rabbit normal IgG (Invitrogen, 31325) in place of primary antibodies. Positive controls were zona-free blastocysts exposed to 20 mM βOHB for 24 h prior to fixing. Images were acquired using a Nikon Eclipse Ti-U inverted fluorescence microscope equipped with a Photometrics Coolsnap HQ_2_ camera with NIS Elements BR 3.0 software. Each embryo was imaged using DAPI, tetramethylrhodamine (TRITC) and fluorescein (FITC) filters. Images were analysed via FIJI image analysis software. TE nuclei were identified by the presence of CDX2; ICM nuclei were identified by an absence of CDX2. The mean CTCF value of five TE nuclei and five ICM nuclei was calculated per embryo, as outlined for NAD(P)H calculations, however ‘background’ areas were selected from the cytoplasmic regions within the embryo. The ‘overall’ CTCF values of each embryo were determined by taking the average CTCF of the same five TE nuclei plus five ICM nuclei (total 10 nuclei). The levels of histone β-hydroxybutyrylation were normalized to DAPI, and the final data presented as the level of β-hydroxybutyrylation normalized to control (untreated) levels within each cell type.

### Embryo transfer

Female F1 mice (8–12 weeks of age) were mated overnight with a vasectomized male to induce pseudopregnancy, which was indicated by the presence of a vaginal plug the following morning. On the morning of Day 4 of culture, all embryos of equivalent morphology (blastocysts, expanded blastocysts and hatching blastocysts) from control and treatment (2 mM βOHB) groups were incubated in 500 µl EmbryoGlue (Vitrolife) at 37°C, 5% O_2_, 6% CO_2_, 89% N_2_, for at least 30 min prior to embryo transfer. Day 4 pseudo-pregnant females were anaesthetized by isoflurane gas, and five control and treatment Day 4 embryos were randomly selected and synchronously transferred surgically to each uterine horn using a polished glass pipette. Control and treatment embryos were transferred contralaterally to avoid preferential implantation bias. Mice were administered Carprofen (5 mg/kg in saline) and Buprenorphine (0.05 mg/kg in saline) via subcutaneous injection immediately following surgery, and oral administration of Buprenorphine (0.1 mg/kg in Nutella) was continued once daily for 72 h post-surgery. Recipient females were killed via cervical dislocation 10 days after embryo transfer surgery, and E14.5 fetal development and/or resorption sites were recorded. Fetal and placental weight, placental diameter, and fetal crown-rump length were recorded, in addition to the morphological grades of limb, ear and eye development (Lane and Gardner, 1994). Fetal sex was determined by dissection of gonads.

### Statistical analyses

All data were tested for normality using the D’Agostino and Pearson omnibus normality test. Proportion data were analysed via a 2 × 5 or 2 × 2 contingency table, with Bonferroni correction for multiple comparisons. One-way or two-way analysis of variance (ANOVA) with Bonferroni correction was used where more than two groups of normal data were compared, as specified, otherwise the Kruskal–Wallis non-parametric test was used with Dunn’s test for multiple comparisons. A two-tailed *t*-test was used to analyse differences between two groups. Between-group differences were analysed within individual time points for data including a time course (morphokinetics and preimplantation βOHB consumption). Statistical significance between data was considered when *P* < 0.05, and all analyses were performed on GraphPad Prism version 6.01 for Windows (www.graphpad.com). Biological replicate and sample sizes are indicated in the relevant figure legends.

### Ethical approval

The University of Melbourne Animal Ethics Committee approved all animal experiments (#10349), which were conducted in accordance with the University’s Animal Care and Use Standards.

## Results

### βOHB retarded preimplantation embryo development and reduced TE cell number

**Figure 1.**
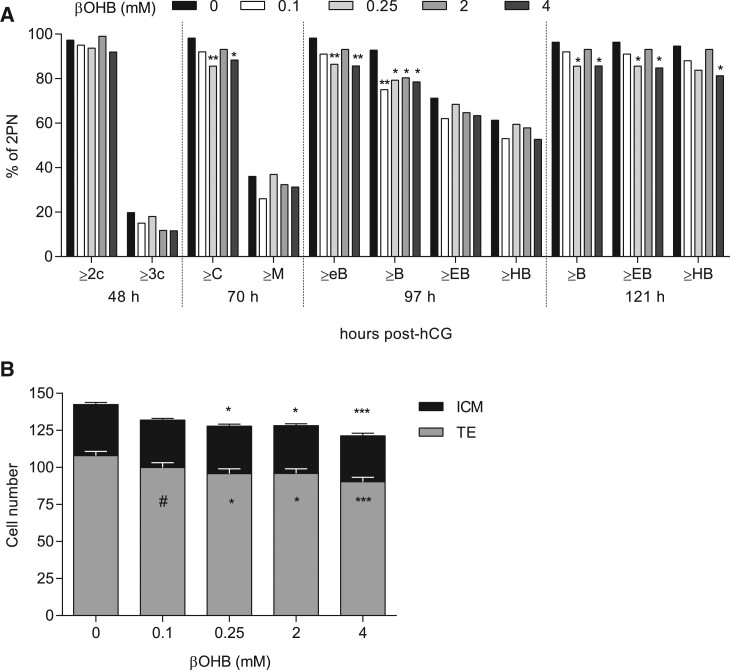
**Effect of β-hydroxybutyrate (βOHB) on preimplantation mouse embryo development.** (**A**) Developmental rates of embryos exposed to βOHB *in vitro*. Data are presented as the proportion of total pronucleate oocytes (2PN) collected that reached or surpassed the indicated developmental stages. N = 100–112 embryos per group, six biological replicates. 2c, 2-cell; 3c, 3-cell; C, compacting embryo; M, morula; eB, early blastocyst; B, blastocyst; EB, expanded blastocyst; HB, hatching blastocyst. Proportion data analysed via 2 × 5 contingency table with Bonferroni *post hoc* analyses. *P <* *0.05, **P <* *0.01, significant compared to control (0 mM). (**B**) Cell number and lineage allocation in Day 5 blastocysts treated with βOHB for 96 h. N = 79–91 blastocysts per group, six biological replicates. Data analysed via Kruskal–Wallis test with Dunn’s correction for multiple comparisons (total cell number, ICM cell number), or one-way ANOVA with Bonferroni correction (TE cell number). Data are presented as mean ± SEM. *P <* *0.05, ***P <* *0.001, ^#^*P *=* *0.06, compared to control (0 mM). Asterisks above the bar represent total cell number, asterisks within the bar represent trophectoderm (TE) cell number.

To determine whether βOHB exposure impacts the rates of preimplantation embryo development and yield, the proportion of embryos reaching or surpassing defined developmental stages was assessed in response to physiological concentrations of βOHB (Fig. 1A). All βOHB concentrations tested (0.1–4 mM) slowed embryonic development compared to unexposed controls. Supplementation with 0.25 mM (*P *<* *0.01) and 4 mM βOHB (*P *<* *0.05) significantly reduced the number of embryos reaching compaction by 70 h post-hCG (Fig. 1A). This developmental retardation was maintained for the remainder of the culture period, as 0.25 mM and 4 mM βOHB exposure yielded fewer early blastocysts (97 h, *P *<* *0.01), blastocysts (97 h and 121 h, *P *<* *0.05), expanded blastocysts (121 h, *P *<* *0.05) and hatching blastocysts (4 mM only, 121 h, *P *<* *0.05) compared to controls (Fig. 1A). Furthermore, a significantly smaller proportion of embryos exposed to 0.1 mM (*P *<* *0.01) and 2 mM βOHB (*P *<* *0.05) reached the blastocyst stage at 97 h post-hCG compared with controls cultured in the absence of βOHB (Fig. 1A).

The number of cells at the blastocyst stage and their allocation to the ICM or TE cell lineages was additionally assessed as this is an established *in vitro* indicator of blastocyst quality (Lane and Gardner, 1997). Total blastocyst cell number was significantly reduced by 0.25 mM (*P *<* *0.05), 2 mM (*P *<* *0.05) and 4 mM βOHB (*P *<* *0.001) compared with unexposed control embryos (Fig. 1B). This decrease was attributable to a reduction in the TE cell number of blastocysts exposed to βOHB (0.25 mM, *P *<* *0.05; 2 mM, *P *<* *0.05; and 4 mM βOHB, *P *<* *0.001). A reduced TE number in 0.1 mM βOHB treated embryos (*P *=* *0.06) compared with controls was also observed but failed to reach statistical significance (Fig. 1B). The number of ICM cells was unaffected by βOHB treatment. βOHB therefore induced developmental delays when assessed at discrete timepoints, which was accompanied by impairments in TE, but not ICM, lineage specification at the blastocyst stage.

### Exposure to 2 mM βOHB slows preimplantation developmental kinetics

**Figure 2.**
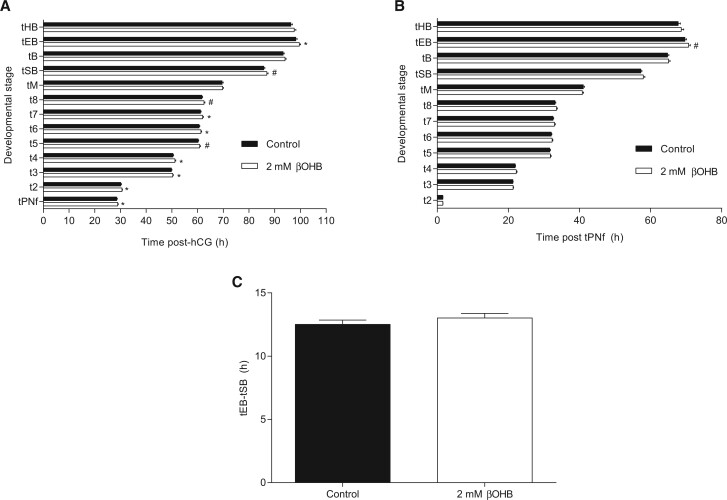
**Morphokinetic development of preimplantation embryos exposed to 2 mM β-hydroxybutyrate (βOHB).** The timing of morphokinetic events is presented as (**A**) time post-hCG injection, or (**B**) time post-pronuclear fading (tPNf). (**C**) Rate of blastocoel expansion (tEB-tSB). N > 79 embryos per group, three biological replicates. Differences between treatments analysed via Student’s *t*-test. Data are presented as mean ± SEM. Asterisks denote statistically significant differences, *P <* *0.05, ^#^*P *<* *0.075, compared to control. Time to: cleavage to 2- to 8-cell stage (t2, t3, t4, t5, t6, t7, t8); morula (tM); start of blastulation (tSB); blastocyst (tB); expanded blastocyst (tEB); hatching blastocyst (tHB).

To precisely identify the onset of βOHB-induced developmental delays at a KD relevant concentration (2 mM), morphokinetic analyses were conducted using a time-lapse incubator (EmbryoScope). Analysis of morphokinetic timings as hours post-hCG revealed exposure to 2 mM βOHB significantly delayed the timing of syngamy (tPNf; *P *<* *0.05) and increased the time to cleavage to 2-cell (t2), 3-cell (t3), 4-cell (t4), 6-cell (t6), 7-cell (t7) and expanded blastocyst (tEB) stages (*P *<* *0.05) compared to controls (Fig. 2A). Furthermore, βOHB appeared to slow development to the 5-cell (t5) (*P *=* *0.054) and 8-cell (t8) stages (*P *=* *0.051), and delay initiation of blastocoel formation (tSB) (*P *=* *0.072; Fig. 2A). Whereas βOHB-treated embryos underwent syngamy (tPNf) on average ∼25 min slower than control embryos, the average developmental delays induced by βOHB became greater as development progressed: βOHB-treated embryos lagged controls by ∼27, 33, 47, 38, 43, 52, 51, 70 and 83 min at the times of t2, t3, t4, t5, t6, t7, t8, tSB and tEB, respectively (Fig. 2A).

To reduce the possible impact of biological variations in mating and insemination times, the timings of key developmental events were additionally expressed as hours post-tPNf (Fig. 2B). When normalized to tPNf, no significant kinetic differences were observed between control and βOHB-treated embryos, however, a non-statistically significant tendency for delayed expansion was maintained (tEB; *P *=* *0.06; Fig. 2B). Furthermore, the trend for the kinetics of βOHB-treated embryos to become increasingly delayed compared with controls was maintained, whereby there was an average developmental lag of ∼0, 5, 19, 10, 15, 24, 23, 41 and 62 min at the times of t2, t3, t4, t5, t6, t7, t8, tSB and tEB, respectively, relative to controls (Fig. 2B). Furthermore, the rate of blastocoel expansion (tEB-tSB) was not significantly different between control and βOHB-treated embryos (*P *=* *0.131; Fig. 2C). Hence the cause of the delayed development in Fig. 2A can be primarily attributed to the significant delay in tPNf, which was significantly impaired in the presence of βOHB. This indicates that apart from syngamy, there were no other stage-specific developmental effects.

### Blastocyst glycolytic metabolism is altered by βOHB exposure in a duration-dependent manner

**Figure 3.**
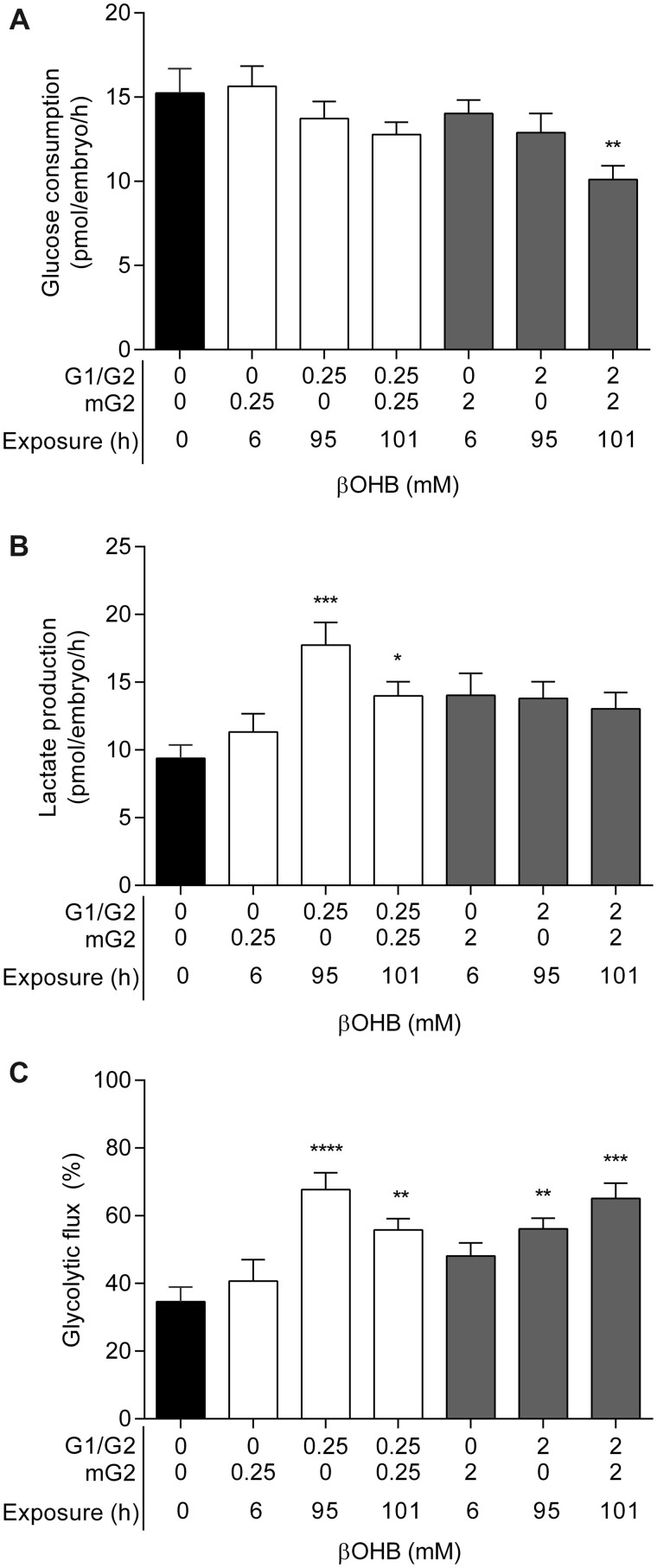
**Glycolytic metabolism of blastocysts exposed to β-hydroxybutyrate (βOHB).** (**A**) Glucose consumption, (**B**) lactate production and (**C**) glycolytic flux of Day 5 blastocysts exposed to 0 mM (control), 0.25 mM or 2 mM βOHB in G1/G2 culture and/or metabolic G2 culture (mG2) for up to 101 h. N = 19–25 blastocysts per group, four biological replicates. Glycolytic flux (%) was calculated as (lactate production × 0.5)/glucose consumption × 100. Data are presented as mean ± SEM. Data were analysed via one-way ANOVA with Bonferroni *post hoc* analysis (glucose uptake), or Kruskal–Wallis test with Dunn’s correction for multiple comparisons (lactate production, glycolytic flux). Asterisks denote statistically significant differences, *P <* *0.05, **P <* *0.01, ***P <* *0.001, ****P <* *0.0001, compared to control (0 mM).

The impact of acute (6 h) and chronic (95–101 h) exposure to standard diet (0.25 mM) and KD (2 mM) concentrations of βOHB on the glycolytic metabolism of Day 5 blastocysts was assessed (Fig. 3). Increasing exposure duration to KD levels of βOHB (2 mM) induced a negative trend in glucose consumption compared with controls, which reached statistical significance at 101 h (*P *<* *0.01; Fig. 3A). A similar duration-dependent reduction in glucose consumption was observed upon 0.25 mM βOHB exposure, however, this did not reach statistical significance (Fig. 3A). The production of lactate was increased by chronic exposure to 0.25 mM βOHB compared with controls (95 h, *P *<* *0.001; 101 h, *P *=* *0.05; Fig. 3B), whereas acute (6 h) exposure to 0.25 mM βOHB had no effect (P > 0.999; Fig. 3B). Likewise, 2 mM βOHB exposure elevated lactate production rates; however, this was not statistically significant compared to controls (6 h, *P *=* *0.189; 95 h, *P *=* *0.126; 101 h, *P *=* *0.221; Fig. 3B). Glycolytic flux was increased by chronic exposure to 0.25 mM βOHB (95 h, *P *<* *0.0001; 101 h, *P *<* *0.01) and 2 mM βOHB (95 h, *P *<* *0.01; 101 h, *P *<* *0.001) compared with controls (Fig. 3C). However, acute (6 h) βOHB exposure did not significantly affect glycolytic flux (0.25 mM βOHB, *P *>* *0.999; 2 mM βOHB, *P *=* *0.205; Fig. 3C).

Consistent with the findings that acute βOHB exposure at physiological concentrations does not impact glycolytic metabolism (Fig. 3), a dose–response analysis found acute (8 h) exposure to 1–4 mM βOHB did not affect blastocyst glucose consumption, lactate production or glycolytic flux compared to controls (Supplementary Fig. S2A–C). However, reduced glucose consumption (*P *<* *0.0001; Supplementary Fig. S2A) and lactate production (*P *<* *0.05; Supplementary Fig. S2B) was observed compared to controls following acute exposure to 8 mM βOHB, representing a supraphysiological level. Therefore, at physiologically relevant concentrations, chronic βOHB exposure induced alterations in glycolytic metabolism, whereas glucose metabolism was suppressed within 8 h when exposed to supraphysiological βOHB levels.

### Preimplantation embryos consume βOHB at all developmental stages via monocarboxylic acid transporters (MCTs)

**Figure 4.**
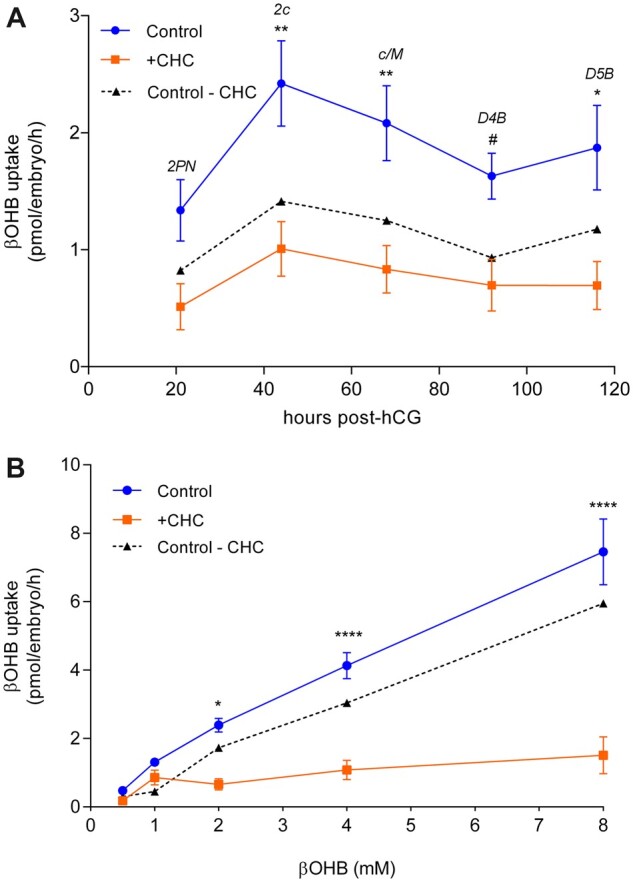
**Characterization of β-hydroxybutyrate (βOHB) consumption by embryos.** (**A**) Rates of βOHB uptake throughout preimplantation development by embryos cultured in 2 mM βOHB alone (control) or with the monocarboxylic acid transporter 1 and 2 inhibitor, α-cyano-4 hydroxycinnamate (CHC, 0.125 mM). Data are presented as mean ± SEM. N = 14–16 metabolic measurements from 70 to 80 embryos per group, four biological replicates. 2PN, pronucleate oocyte; 2c, 2-cell; c/M, compacting/morula stage; D4B, Day 4 blastocyst; D5B, Day 5 blastocyst. (**B**) Rates of βOHB uptake by D5Bs exposed to 0.5–8 mM βOHB alone (control) or with CHC (0.125 mM). Data are presented as mean ± SEM. N = 38–81 metabolic measurements from 190 to 405 blastocysts per control group; 15 metabolic measurements from 75 blastocysts per +CHC group; 15 biological replicates. Differences between control and +CHC were analysed by two-way ANOVA with Bonferroni correction for multiple comparisons. Asterisks denote statistically significant differences, *P <* *0.05, **P <* *0.01, ****P <* *0.0001, ^#^*P *<* *0.08. ‘Control—CHC’ represents levels of MCT-facilitated βOHB uptake.

To determine whether preimplantation embryos can metabolize βOHB, a novel enzyme-coupled fluorometric assay was developed for the non-invasive quantification of βOHB uptake. The rates of βOHB consumption by 2PN, 2-cell, compacting/morula-stage, Day 4 and Day 5 blastocysts exposed to KD-relevant levels (2 mM βOHB) were assessed, and revealed that embryos consumed βOHB at all developmental stages (Fig. 4A). There was a peak in βOHB uptake rates at the 2-cell (∼2.4 pmol/embryo/h) and compacting/morula stages (∼2.1 pmol/embryo/h), with lower rates of uptake by 2PNs (∼1.3 pmol/embryo/h), Day 4 blastocysts (∼1.6 pmol/embryo/h) and Day 5 blastocysts (∼1.9 pmol/embryo/h) (Fig. 4A).

To determine whether MCTs are the mechanism by which βOHB consumption is facilitated in preimplantation embryos, a broad MCT1/2 inhibitor, CHC (Halestrap and Price, 1999), was included in metabolic culture medium. The addition of 0.125 mM CHC (Supplementary Fig. S1) reduced βOHB uptake at all developmental stages, reaching significance at the 2-cell (*P *<* *0.05), compacting/morula stages (*P *<* *0.05), and Day 5 blastocyst stage (*P* < 0.05), approaching significance at the Day 4 blastocyst (*P *=* *0.076), but not at the 2PN stage (*P *=* *0.159; Fig. 4A). Interestingly, CHC did not completely inhibit βOHB consumption, which remained at approximately ∼0.75 pmol/embryo/h throughout development in the presence of the transport inhibitor (Fig. 4A).

The characteristics of βOHB uptake were further explored in Day 5 blastocysts exposed to increasing βOHB concentrations (0.5–8 mM βOHB) with or without CHC (0.125 mM). βOHB consumption increased linearly with substrate availability (Fig. 4B). The addition of CHC significantly reduced uptake at 2 mM (*P *<* *0.05), 4 mM (*P *<* *0.0001) and 8 mM (*P *<* *0.0001). However, at lower βOHB concentrations CHC did not reduce βOHB uptake rates [0.5 mM (*P *> 0.99) and 1 mM (*P *> 0.99)] (Fig. 4B).

### Redox state is not altered by βOHB exposure

**Figure 5.**
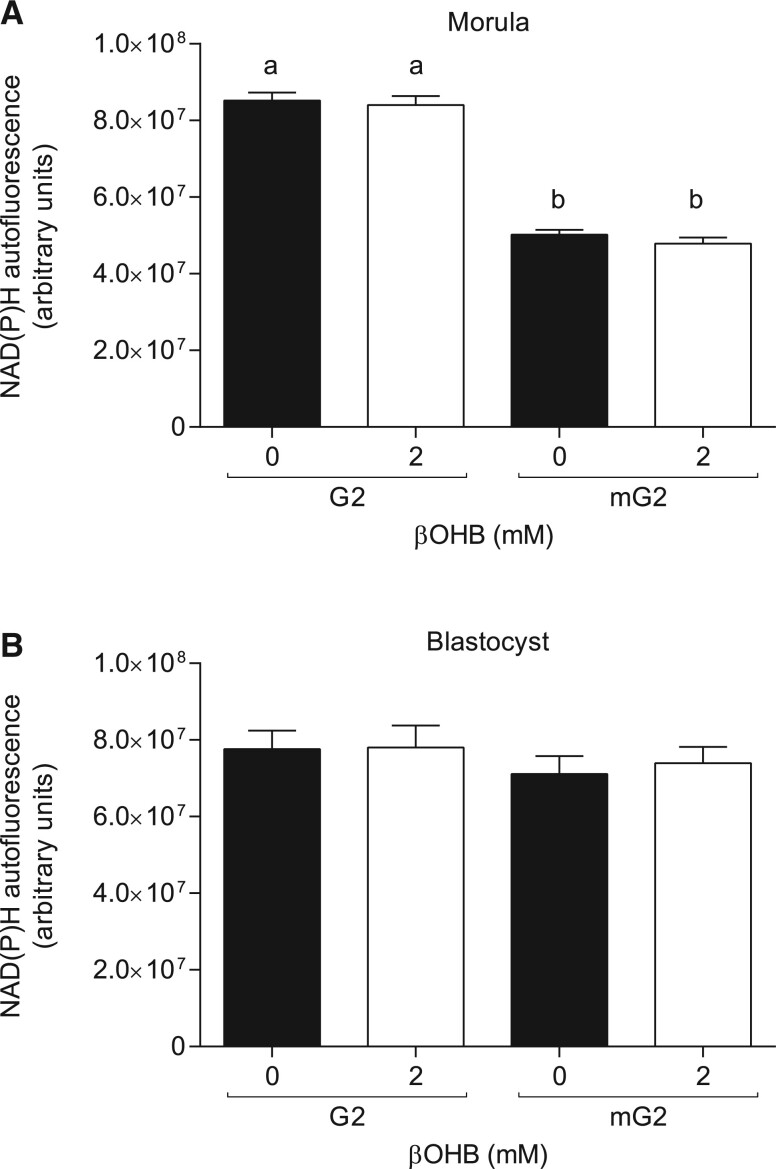
**Redox state of morulae and Day 5 blastocysts following exposure to β-hydroxybutyrate (βOHB).** NAD(P)H autofluorescence of (**A**) morulae and (**B**) Day 5 blastocysts exposed to 0 mM (control) or 2 mM βOHB for 20 min in standard G2 medium, or metabolic G2 medium (mG2, 0.5 mM glucose, no lactate or pyruvate). N = 29–30 embryos per group, three biological replicates. Data are presented as mean ± SEM. Differences between control and treatment were analysed via two-way ANOVA with Bonferroni *post hoc* analysis. Statistically significant differences are denoted by different letters; ^a,b^*P *<* *0.0001.

The cytosolic redox state of morulae (Fig. 5A) and blastocysts (Fig. 5B) exposed to 0 mM or 2 mM βOHB in standard G2 medium or metabolic G2 medium was assessed via imaging of NAD(P)H autofluorescence. Exposure to 2 mM βOHB for a short duration (20 min) had no detectable impact on the cytosolic redox state of morulae or blastocysts compared with untreated controls, regardless of exposure in G2 medium or metabolic G2 medium (Fig. 5). However, significantly lower NAD(P)H autofluorescence was detected in morulae exposed to metabolic G2 medium compared to those in standard G2 medium (*P *<* *0.0001; Fig. 5A), indicating that a 20 min exposure window was sufficient to induce a detectable redox shift due to the removal of lactate and pyruvate from the culture system. Unlike morulae, blastocysts did not exhibit a reduction in NAD(P)H autofluorescence due to exposure to metabolic G2 (Fig. 5B).

### H3K27bhb is elevated by supraphysiological, but not physiological βOHB concentrations

**Figure 6.**
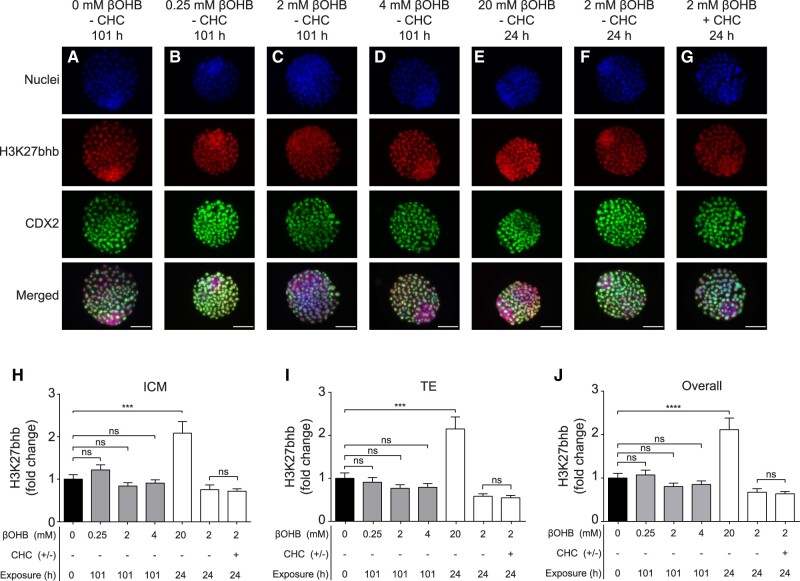
**Impact of β-hydroxybutyrate (βOHB) on blastocyst histone 3 lysine 27 β-hydroxybutyrylation (H3K27bhb).** Embryos were exposed from the 2PN until Day 5 blastocyst stage (101 h) in (**A**) 0 mM, (**B**) 0.25 mM, (**C**) 2 mM and (**D**) 4 mM βOHB, or from the Day 4 until Day 5 blastocyst stage (24 h) in (**E**) 20 mM, or (**F, G**) 2 mM βOHB. (**G**) Embryos were additionally cultured with the MCT1/2 inhibitor, α-cyano-4-hydroxycinnamate (CHC, 0.125 mM) for the duration of βOHB exposure. H3K27bhb levels were quantified in (**H**) inner cell mass (ICM) cells and (**I**) trophectoderm (TE) cells. The ‘overall’ level of H3K27bhb presented in (**J**) is the average of ICM + TE results. Data are presented as mean fold change from control ± SEM. Differences between control (0 mM βOHB, black bars) and treatments were analysed via Kruskal–Wallis test with Dunn’s test for multiple comparisons. Asterisks denote statistically significant differences. ***P <* *0.001, ****P <* *0.0001, ns, not significant. N = 42–47 blastocysts per group (A–D, F, G), n = 21 blastocysts (E), from three independent biological replicates.

The impact of βOHB on H3K27bhb was assessed via immunofluorescence within the ICM and TE cell lineages of βOHB-exposed blastocysts. Representative images of embryos stained for H3K27bhb, CDX2 and nuclei (Hoechst 33258) following treatment with or without βOHB and CHC are presented in Fig. 6A–G. H3K27bhb was detected in unexposed (0 mM βOHB) control blastocysts (Fig. 6A). Exposure to 20 mM βOHB for 24 h (positive control) (Sangalli *et al.*, 2022) significantly elevated H3K27bhb levels ∼2-fold compared with controls in both the ICM and TE (*P *<* *0.001, Fig. 6H and I) and overall (*P *<* *0.0001, Fig. 6J). However, exposure to physiologically relevant βOHB concentrations (0.25–4 mM) for 101 h did not impact H3K27bhb levels in the ICM, TE or overall compared with controls (Fig. 6H–J). Similarly, there was no significant difference in H3K27bhb levels between embryos exposed to 2 mM βOHB for 24 h with or without CHC (Fig. 6H–J).

### Post-transfer viability and placental and fetal development are compromised by preimplantation exposure to βOHB

The impact of preimplantation exposure for 72 h to KD levels of βOHB (2 mM) on the post-transfer viability of embryos was assessed following synchronous transfer of Day 4.5 blastocysts to standard-fed pseudopregnant female recipients. Analysis of E14.5 fetal and placental development revealed a significant impairment of post-transfer embryonic viability following preimplantation βOHB exposure, with females more severely impacted than males (Table II). βOHB-treated embryos had a 50% lower rate of implantation per transfer (*P *<* *0.05) and fetal development per transfer (*P *<* *0.05) compared with controls. However, the rate of fetal development per implantation was unaffected by βOHB (*P *=* *1.00). The combined (all fetus) data indicated placental diameter (*P *<* *0.05) and fetal crown-rump (C-R) length (*P *<* *0.01) were significantly smaller in embryos cultured with βOHB, and there was an apparent trend for reduced fetal limb morphological grade by βOHB compared with controls (*P *=* *0.075). Stratification of data by sex revealed female βOHB-treated embryos weighed less (*P *<* *0.05), had a shorter C-R length (*P *<* *0.05), and tended to have a higher ear morphological grade (*P *=* *0.07) by E14.5 compared with controls, whereas amongst male fetuses there was an apparent trend for reduced C-R length only (*P *=* *0.07; Table II). There were no significant differences in eye morphological grade or estimated fetal age between control and treatment fetuses. Similarly, βOHB-exposure did not impact placental weight, the fetal/placental weight ratio, the rate of resorptions per transfer, nor the sex ratio, compared to controls (Table II).

**Table II:** Fetal and placental development following Day 4 synchronous mouse blastocyst transfer after *in vitro* culture from 2 pronucleate oocyte stage with 2 mM β-hydroxybutyrate (βOHB).

Parameter	Control		2 mM βOHB	
	Mean or %	SEM or (n/N)	Mean or %	SEM or (n/N)
**No. embryos transferred**	46		45	
**Implantation per transfer**	59%	(27/46)	31%	(14/45)^*^
**Fetal development per transfer**	48%	(22/46)	24%	(11/45)^*^
**Fetal development per implantation**	81%	(22/27)	79%	(11/14)
**Resorption per transfer**	11%	(5/46)	7%	(3/45)
**Sex ratio (% male)**	36%	(8/22)	45%	(5/11)
**Fetal weight (mg)**				
Combined	207.69	±6.07	201.45	±14.35
Female	207.90	±9.04	179.12	±14.46^*^
Male	207.33	±6.14	228.26	±22.12
**Placental weight (mg)**				
Combined	97.69	±5.43	90.28	±8.91
Female	98.55	±7.26	85.90	±11.88
Male	96.09	±8.36	95.54	±14.58
**Fetal/placental weight ratio**				
Combined	2.27	±0.13	2.45	±0.30
Female	2.24	±0.17	2.28	±0.35
Male	2.32	±0.23	2.67	±0.54
**Placental diameter (mm)**				
Combined	8.54	±0.12	7.73	±0.37^*^
Female	8.47	±0.15	7.67	±0.39
Male	8.65	±0.21	7.80	±0.72
**Crown-rump length (mm)**				
Combined	11.81	±0.17	10.93	±0.23^**^
Female	11.86	±0.24	10.74	±0.38^*^
Male	11.71	±0.18	11.16	±0.19^#^
**Limb morphological grade**				
Combined	14.93	±0.07	14.64	±0.21^#^
Female	14.89	±0.11	14.42	±0.37
Male	15.00	±0.00	14.90	±0.10
**Eye morphological grade**				
Combined	14.86	±0.07	14.64	±0.24
Female	14.93	±0.07	14.33	±0.42
Male	14.75	±0.16	15.00	±0.00
**Ear morphological grade**				
Combined	14.80	±0.08	14.91	±0.06
Female	14.75	±0.11	14.83	±0.11^#^
Male	14.88	±0.13	15.00	±0.00
**Estimated fetal age**				
Combined	14.84	±0.04	14.70	±0.17
Female	14.82	±0.05	14.43	±0.30
Male	14.88	±0.06	14.97	±0.03

Differences between treatments analysed via unpaired *t*-test or Mann–Whitney *U* test depending on normality of distributions, or via 2 × 2 contingency table for proportion data. N = 9 replicates of 5 embryos transferred per horn (with the exception of one recipient in the control that received 6 blastocysts).

Asterisks indicate statistically different from control.

* *P *<* *0.05

** *P *<* *0.01

# *P* < 0.08.

## Discussion

Consumption of a KD by healthy reproductive-aged women is increasing in popularity, however, there is insufficient evidence supporting its safety as a gestational or pre-gestational diet. Although the KD appears to improve fertility outcomes in women with PCOS by correcting hormonal and metabolic imbalances (Mavropoulos *et al.*, 2005; Gupta *et al.*, 2017; Alwahab *et al.*, 2018; Paoli *et al.*, 2020), rodent studies have indicated negative offspring developmental effects of a gestational KD (Sussman *et al.*, 2013a,b, 2015; Wojciech *et al.*, 2022) and supraphysiological *in vitro* βOHB exposure (Zusman *et al.*, 1987; Moley *et al.*, 1994). The present study is the first to explore the underlying mechanisms by which βOHB at physiologically relevant exposures during *in vitro* preimplantation development may impact offspring physiology, development and viability, by utilizing a healthy mouse model. Data reveal that embryonic βOHB exposure and utilization induces aberrant preimplantation development and reduces post-transfer viability in mice. Altered blastocyst metabolism and aberrations specific to the TE cell lineage are possibly related to implantation and placental insufficiencies post-transfer, subsequently restricting fetal growth, with female offspring most severely impacted.

### Preimplantation βOHB exposure impairs embryonic development and post-transfer viability

Preimplantation embryos exposed to physiological concentrations (≤4 mM) of βOHB *in vitro* experienced significantly delayed development compared with untreated controls, indicating poorer viability. Developmental delays were first identified at compaction when morphology was assessed at discrete timepoints, while the utilization of time-lapse microscopy revealed development was delayed as early as syngamy. In contrast, Moley *et al.* (1994) previously reported exposures to ≥16 mM βOHB retarded mouse preimplantation development, however time-lapse microscopy was not utilized, and no effect on development was observed by supplementation with 8 mM βOHB. This discrepancy may be due to the earlier onset of βOHB exposure at syngamy in the present study compared with the late 2-cell stage in the study by Moley and colleagues (Moley *et al.*, 1994). This suggests developmental events prior to embryonic genome activation, occurring at the 1- to 2-cell stage in mice (Eckersley-Maslin *et al.*, 2018), may have increased susceptibility to βOHB that lead to significant and prolonged disruptions to physiology and developmental processes.

Developmental delays induced by βOHB culminated in blastocysts with significantly fewer cells, that notably was due to a specific reduction in TE cell number. Low total cell numbers have been correlated with reduced fetal developmental rates post-transfer (Lane and Gardner, 1997), thus further indicating that βOHB exposure may impair viability. However, the TE-specific reduction in cell number suggests implantation and placental development may be impaired, given the TE lineage forms the placenta. Indeed, this was supported by embryo transfer experiments in the present study whereby preimplantation βOHB exposure reduced implantation rates by 50%. Fetal development per implantation and the rate of fetal resorption was unaffected by βOHB exposure, indicating βOHB does not induce loss of pregnancy, but rather impairs the implantation process and/or placental formation. Indeed, fetuses developing from βOHB-exposed embryos had smaller placental diameter and restricted fetal growth compared with unexposed controls. It is therefore plausible that, in addition to aberrant implantation, elevated ketone exposure may perturb placental formation and cause functional insufficiencies leading to the restriction of fetal nutrient provision. Fetal growth restriction can in turn predispose offspring to negative long-term health consequences, such as hypertension, kidney disease, and diabetes (reviewed by Doan *et al.* (2022)). Assessments of placentae from diabetic rodent pregnancies, during which time diabetic ketoacidosis and elevated ketone levels are common (Laffel, 1999), have identified placental structural abnormalities including impaired spongiotrophoblast cell differentiation and small labyrinth and junctional layers (Salbaum *et al.*, 2011). Future histological analyses of placentae derived from βOHB-exposed embryos will be of immense value to further elucidate the mechanisms of βOHB aberration. Further, rodent studies have identified a maternal gestational KD to produce smaller offspring on embryonic Day 17.5 (Sussman *et al.*, 2013b) and postnatal Day 2 (Wojciech *et al.*, 2022) compared to offspring from standard fed mothers. Together, these data suggest elevated gestational ketone levels may contribute to developmental and functional insufficiencies of the placenta that affects fetal growth. βOHB exposure at KD-relevant levels is therefore detrimental to the development and viability of embryos, with negative effects likely mediated through aberrant physiology and developmental programming of the TE cell lineage.

### Mechanisms underpinning the viability-perturbing effects of βOHB

Exogenous nutrients impact embryonic metabolism and epigenetic state, and thereby act as signals to prepare offspring for post-partum nutrient availability. Misalignment between gestational and post-partum nutritional levels contributes to metabolic disorders and pre-disposes offspring to long-term health aberrations, as described by DOHaD (Barker and Osmond, 1986; Hales and Barker, 1992; Fleming *et al.*, 2015). To elucidate how and why βOHB negatively impacts mouse embryonic development and viability, it is therefore necessary to understand how βOHB is utilized by embryos, and how it affects physiological and epigenetic processes that may contribute to long-term developmental outcomes.

Significantly, the profile of βOHB consumption throughout preimplantation development, reported here for the first time, indicates βOHB is utilized oxidatively by embryos. Prior to compaction, embryos have a low capacity for glycolytic metabolism and primarily utilize pyruvate for oxidative phosphorylation. The identified βOHB uptake profile bears a striking resemblance to that of pyruvate consumption by preimplantation mouse embryos, with comparable consumption rates observed at the 2PN (∼1.3 pmol βOHB/embryo/h versus ∼0.76–1.3 pmol pyruvate/embryo/h), 2-cell (∼2.4 pmol βOHB/embryo/h versus ∼1.2–2 pmol pyruvate/embryo/h) and 8-cell to compacting/morula stages (∼2.1 pmol βOHB/embryo/h versus ∼1.3–2.3 pmol pyruvate/embryo/h) (Leese and Barton, 1984; Gardner and Leese, 1986). The strong resemblance between βOHB and pyruvate utilization is therefore significant, albeit indirect, evidence that βOHB is metabolized oxidatively by preimplantation embryos, however, future analyses of ATP flux and oxygen consumption will be important to confirm this.

Throughout development, preimplantation embryos consume βOHB via MCT1 and 2, confirmed by the addition of the MCT inhibitor CHC. Both MCT1 and MCT2 have specifically been detected on the apical membrane of TE cells, whereas they are absent from the TE cell basal membrane and from ICM cells (Jansen *et al.*, 2006). Significantly, MCT1/2-facilitated uptake of βOHB by blastocysts occurred primarily at KD-relevant concentrations (≥2 mM βOHB), whereas at standard diet levels (≤1 mM βOHB) βOHB consumption was minimal. The observed consumption of βOHB at low rates even in the presence of CHC can be attributed to uptake via passive diffusion because of the high plasma membrane permeability of βOHB. The uptake of βOHB by other MCT isoforms (such as MCT3 or MCT4) is improbable given the absence of their mRNA and protein in mouse embryos (MCT3) (Hérubel *et al.*, 2002; Jansen *et al.*, 2006) or extremely low affinity for βOHB (MCT4, Km_βOHB_ = 130 mM) (Halestrap, 2013). Under normal physiological conditions of very low βOHB availability (<1 mM βOHB), preimplantation embryos would therefore have minimal rates of βOHB consumption facilitated by simple transmembrane diffusion. At physiologically relevant concentrations (≤4 mM βOHB), the TE cell lineage, but not the ICM, is therefore able to consume and be affected by βOHB, supporting the TE-specific effects observed on embryo development and viability including reduced TE cell number and post-transfer implantation aberrations.

Chronic βOHB exposure perturbed the glycolytic metabolism of blastocysts, whereby lower glucose consumption and increased glycolytic flux was observed. The significant decrease in glucose consumption was likely accompanied by reduced glucose-derived carbon flux through the pentose phosphate pathway (PPP) and production of biosynthetic precursors (Gardner and Harvey, 2015), culminating in reduced TE cell number and function, potentially underpinning implantation failure. βOHB at supraphysiological exposures (32 mM) has previously been reported to reduce PPP activity and compromise ribose moiety synthesis in *ex vivo* Day 9 mouse conceptuses (Hunter *et al.*, 1987) likely reducing cell proliferation. In the human, the grade of the TE appears a strong indicator of blastocyst viability (Ahlström *et al.*, 2011). The rate of glycolytic flux has previously been reported at ∼30–50% in mouse embryos developed *in vivo* (Gardner and Leese, 1990; Lane and Gardner, 1998) and in ‘good’ quality mouse blastocysts cultured *in vitro* in low oxygen (5% O_2_) conditions. ‘Poor’ quality mouse blastocysts exhibit a high glycolytic flux within the range of ∼59–78% (Wale and Gardner, 2012; Lee *et al.*, 2015), which is associated with impaired post-transfer viability, ascribed to the premature utilization of endogenous glycogen (Lane and Gardner, 1996). Chronic βOHB exposure significantly increased glycolytic flux to ∼56–68%; a range comparable to ‘poor’ quality embryos (Wale and Gardner, 2012; Lee *et al.*, 2015) that suggests βOHB-exposed embryos may lose their ability to regulate metabolism in a manner compatible with ongoing and/or healthy development.

Embryos in the present study exposed to physiologically relevant βOHB concentrations (≤4 mM) experienced no change in β-hydroxybutyrylation levels (H3K27bhb) compared to controls. This is despite previous reports that comparable βOHB concentrations (2–6 mM) can induce a dose–responsive increase in H3K9bhb in bovine fibroblasts and cumulus cells, detected via immunofluorescence (Sangalli *et al.*, 2022). Supraphysiological βOHB exposure (20 mM), however, did increase H3K27bhb levels in both the TE and ICM cell lineages, despite the ICM not possessing the necessary transporters for βOHB uptake (Jansen *et al.*, 2006). This reinforces that βOHB is partially consumed via simple diffusion and can target both cell lineages at supraphysiological concentrations. These findings demonstrate a capability for mouse blastocysts to regulate H3K27bhb levels in response to extreme βOHB exposure, however this starvation-responsive epigenetic modification (Xie *et al.*, 2016) is unlikely to have contributed to the metabolic and morphological developmental adaptations observed here. Other potential epigenetic targets of βOHB, such as histone acetylation, could alternatively be impacted by βOHB exposure, however this remains to be examined.

In addition to its role as a histone β-hydroxybutyrylation substrate, βOHB can increase histone acetylation via its function as an endogenous class I HDAC inhibitor (Newman and Verdin, 2017). In cells with high rates of aerobic glycolysis, such as some cancers, βOHB that is not/cannot be metabolized oxidatively instead accumulates in the nucleus where its HDAC inhibiting activity down-regulates GLUT1 expression and promotes cell death and apoptosis pathways (Li *et al.*, 2006; Donohoe *et al.*, 2012; Rodrigues *et al.*, 2017). This metabolism-dependent epigenetic function of βOHB has been described as the ‘βOHB paradox’ (Rodrigues *et al.*, 2017). Results from the present study provide indirect evidence of the existence of the ‘βOHB paradox’ in TE cells and indicate that reduced glucose consumption and TE cell number may be regulated on an epigenetic level. Firstly, acute βOHB exposure did not alter the redox state, indicating changes in availability of the glycolytic enzyme co-factor, NAD^+^, was not a mechanism that contributed to down-regulation of glucose consumption. Secondly, chronic βOHB exposure was necessary to induce changes in glucose consumption and TE cell number. This suggests there may be a gradual accumulation of excess/unmetabolized βOHB within the nuclei of TE cells over time, that could epigenetically down-regulate GLUT1 expression and promote apoptotic pathways, as per the ‘βOHB paradox’. In addition to alternative epigenetic modifications, further analyses of gene expression and apoptosis are warranted.

Following embryo transfer to healthy, standard-fed recipient females, βOHB-exposed mouse embryos produced smaller fetuses than control embryos, suggesting a developmental programming effect. Notably, this effect appeared to be sex-specific, as βOHB-treated female fetuses weighed less and had more rapid ear development compared to female control fetuses, while similar developmental alterations were not observed amongst males. Past assessments of the impact of a gestational KD on fetal development have not accounted for fetal sex (Sussman *et al.*, 2013b), however, post-natal behavioural analyses have identified female pups, but not males, from KD-fed mothers to have increased hyperactivity and faster reflexes (hindlimb placing) compared to females from standard-fed mothers (Sussman *et al.*, 2015; Wojciech *et al.*, 2022). Female-specific physiology may contribute to these sex-specific developmental programming events. Prior to X chromosome inactivation (XCI), an epigenetic process initiated in the TE cell lineage during implantation, female embryos have two active X-chromosomes carrying, among others, the gene for the glycolytic enzyme, glucose-6-phosphate dehydrogenase (G6PD). Higher G6PD expression renders female embryos more glycolytic than males (Gardner *et al.*, 2010) and may consequently increase their susceptibility to the negative epigenetic impacts of βOHB, as per the ‘βOHB paradox’ (Rodrigues *et al.*, 2017). XCI regulates the expression of ∼600 X-linked genes and over ∼2900 autosomal genes in mouse and bovine embryos, respectively (Kobayashi *et al.*, 2006; Bermejo-Alvarez *et al.*, 2010; Gardner *et al.*, 2010), and aberrations in this epigenetic process could therefore underpin the female-targeted developmental programming effects of βOHB. The consequences of peri-conceptional/gestational βOHB exposure for female reproductive potential in subsequent generations will be an important future consideration.

Here we have provided a comprehensive analysis of the impact of βOHB on preimplantation embryo development, physiology and viability, however, there are several limitations that should be considered when interpreting results. Firstly, the *in vitro* model utilized to assess the impact of βOHB on embryo development does not mimic the *in vivo* female reproductive tract and therefore does not consider other possible factors changed by KD consumption, such as glucose, protein and hormonal levels. Secondly, we assessed βOHB exposure specifically during preimplantation embryo development, and have not considered how prolonged exposure during gamete development, fertilization and post-implantation fetal development may further contribute to developmental and physiological parameters. Finally, the concentrations of βOHB utilized in these experiments were modelled on human and rodent blood/serum levels because the oviduct and uterine concentrations are unknown and could possibly be an under- or over-estimation of true βOHB concentrations within reproductive fluid. To address some of these limitations, current experiments in our laboratory are underway to assess the impact of a maternal KD on *in vivo* blastocyst development.

## Conclusion

This study reveals that preimplantation embryos are capable of consuming βOHB as an oxidative energy source; however, its utilization is detrimental for mouse preimplantation development and post-transfer viability. Exposure to KD-relevant βOHB concentrations *in vitro* significantly reduced TE cell number at the blastocyst stage, and further delayed morphokinetic development and increased glycolytic flux, indicative of poor viability. Through TE-mediated metabolic mechanisms, βOHB may affect developmental programming of mouse embryos, reduce post-transfer implantation potential and restrict placental and fetal development, particularly in female offspring. Further investigation to elucidate if and how βOHB impacts gamete development, and fetal development in late-stage gestation and post-partum is required. The consumption of diets which increase βOHB levels, such as a KD, by females without underlying hormonal/metabolic aberrations may therefore affect preimplantation embryo development and viability and may not be appropriate for periconceptional consumption. Further research to confirm these observations in humans is warranted.

## Supplementary Material

deac153_Supplementary_Figure_S1

deac153_Supplementary_Figure_S2

## Data Availability

The data underlying this article are available in the article and in the online supplementary material.
