# Supplementary material for: β-hydroxybutyrate reduces blastocyst viability via trophectoderm-mediated metabolic aberrations in mice
Source: Hum Reprod. 2022 Jul 20;37(9):1994–2011. doi: 10.1093/humrep/deac153 (PMC9433850; doi:10.1093/humrep/deac153)
Supplement: deac153_Supplementary_Figure_S1 [file deac153_supplementary_figure_s1.pdf]

Human Reproduction, pp. 1–1, 2022
https://doi.org/10.1093/humrep/deac153

human reproduction

SUPPLEMENTARY DATA

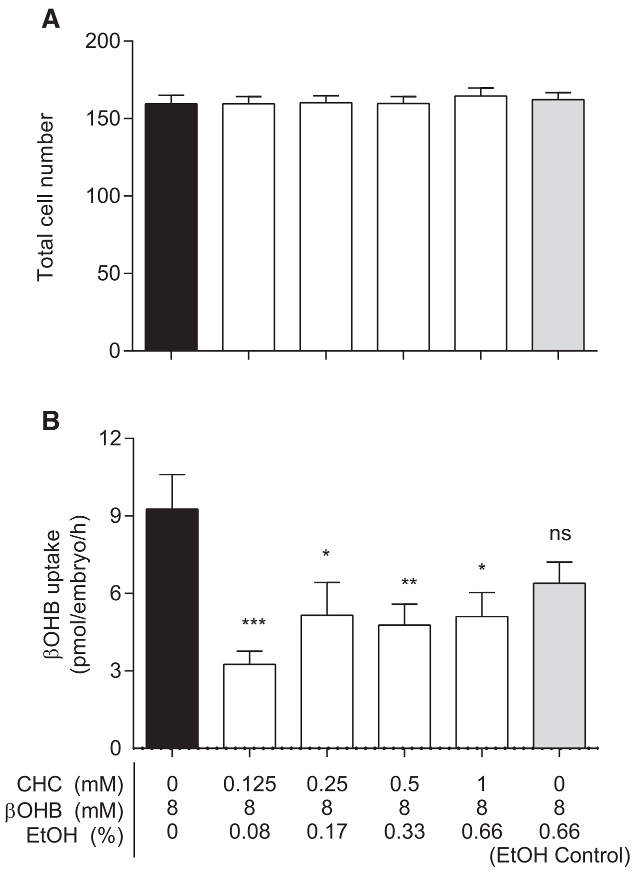

Supplementary Figure S1. Concentration optimization of the monocarboxylate transporter (MCT) 1/2 inhibitor alpha-cyano-4-hydroxycinnamate (CHC). (**A**) Total cell number of blastocysts used for CHC concentration optimization. (**B**) Rates of βOHB uptake by Day 5 blastocysts exposed to 8 mM βOHB with or without CHC in an ethanol (EtOH) vehicle. N = 12–15 measurements per group, from 60 to 75 blastocysts per group. Data are mean ± SEM. Data analysed via Kruskal–Wallis test with Dunn's test for multiple comparisons. Asterisks represent statistical significance compared to control (8 mM βOHB, no CHC or EtOH). *$P < 0.05$, **$P < 0.01$, ***$P < 0.001$, ns, not significant.
