# Supplementary material for: β-hydroxybutyrate reduces blastocyst viability via trophectoderm-mediated metabolic aberrations in mice
Source: Hum Reprod. 2022 Jul 20;37(9):1994–2011. doi: 10.1093/humrep/deac153 (PMC9433850; doi:10.1093/humrep/deac153)
Supplement: deac153_Supplementary_Figure_S2 [file deac153_supplementary_figure_s2.pdf]

Human Reproduction, pp. 1–1, 2022
https://doi.org/10.1093/humrep/deac153

human reproduction

SUPPLEMENTARY DATA

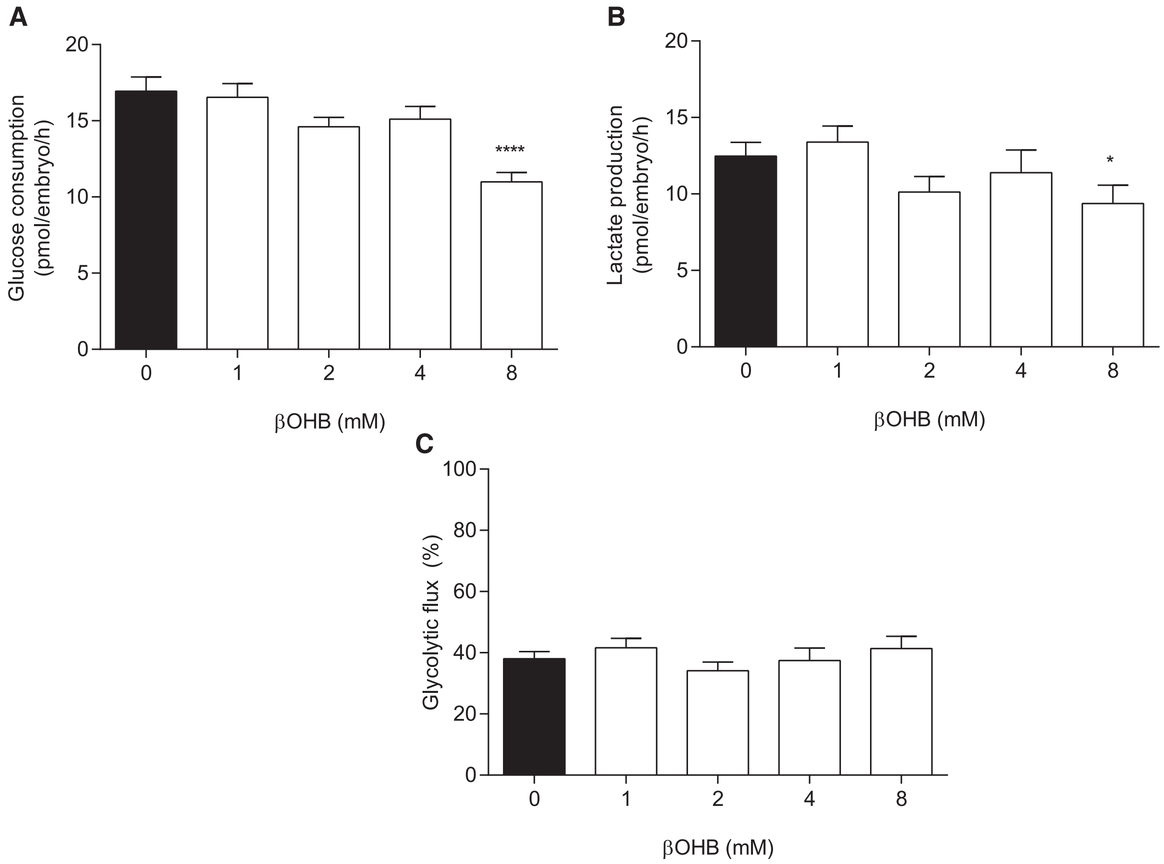

Supplementary Figure S2. Glycolytic metabolism of blastocysts exposed to β-hydroxybutyrate (βOHB) for just 8 h. (**A**) Glucose consumption, (**B**) lactate production and (**C**) glycolytic flux of Day 5 blastocysts exposed to 0 mM (control), 1, 2, 4 and 8 mM βOHB in metabolic G2 culture for 8 h. N = 23–26 blastocysts per group, three biological replicates. Data are presented as mean ± SEM. Data were analysed via one-way ANOVA with Bonferroni *post hoc* analysis (glucose uptake), or Kruskal–Wallis test with Dunn's correction for multiple comparisons (lactate production, glycolytic flux). Asterisks denote statistically significant differences compared to control (0 mM), *$P < 0.05$, ****$P < 0.0001$.
